# Supplementary material for: The causal role of circulating inflammatory markers in osteoporosis: a bidirectional Mendelian randomized study
Source: Front Immunol. 2024 Jul 18;15:1412298. doi: 10.3389/fimmu.2024.1412298 (PMC11291241; doi:10.3389/fimmu.2024.1412298)
Supplement: Supplementary file 1 [file DataSheet_1.zip › supplementary material/OSTEOPOROSIS-CIM/GCST90274771.tsv.gz_leave_one_out.pdf]

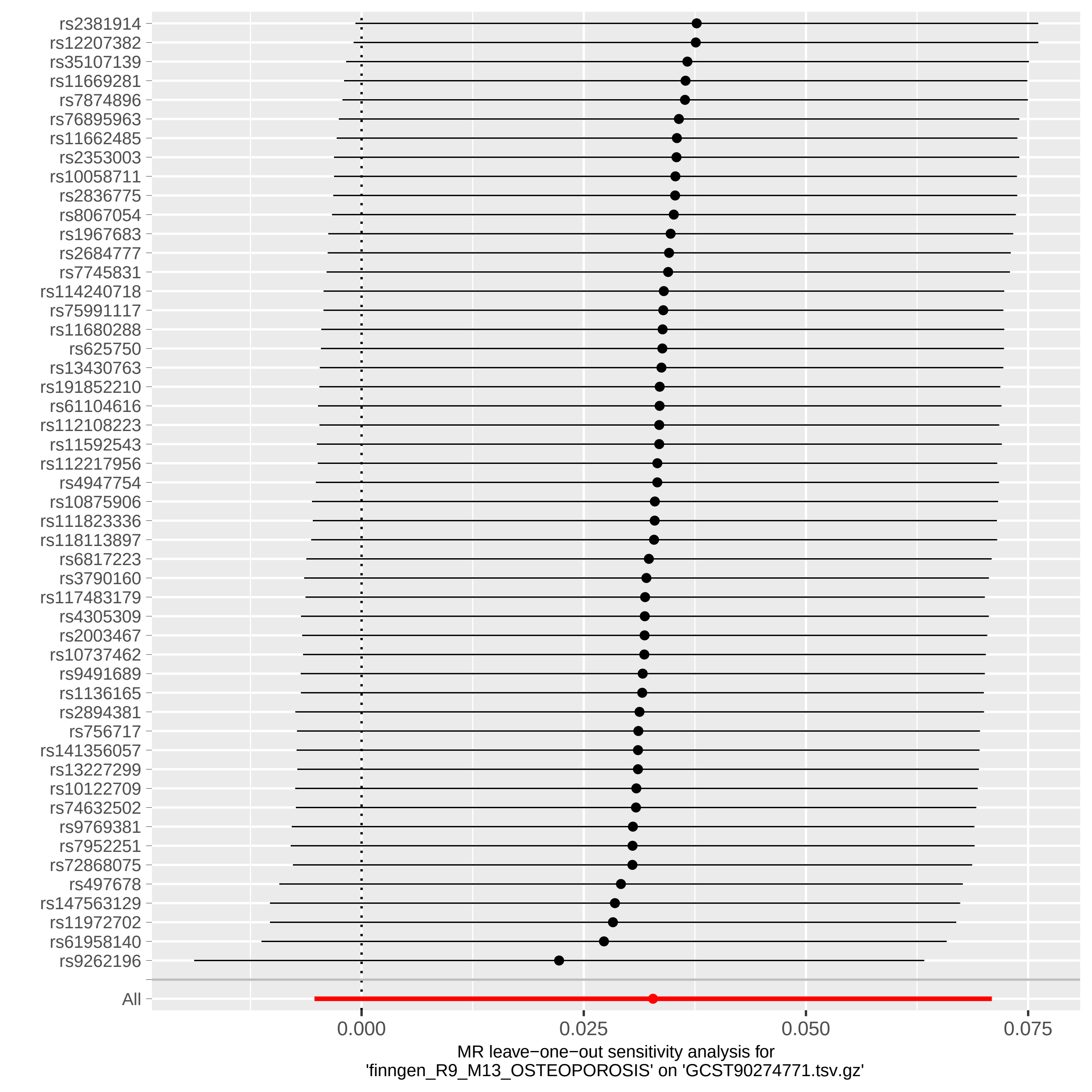
rs2381914
rs12207382
rs35107139
rs11669281
rs7874896
rs76895963
rs11662485
rs2353003
rs10058711
rs2836775
rs8067054
rs1967683
rs2684777
rs7745831
rs114240718
rs75991117
rs11680288
rs625750
rs13430763
rs191852210
rs61104616
rs112108223
rs11592543
rs112217956
rs4947754
rs10875906
rs111823336
rs118113897
rs6817223
rs3790160
rs117483179
rs4305309
rs2003467
rs10737462
rs9491689
rs1136165
rs2894381
rs756717
rs141356057
rs13227299
rs10122709
rs74632502
rs9769381
rs7952251
rs72868075
rs497678
rs147563129
rs11972702
rs61958140
rs9262196
All
0.000
0.025
0.050
0.075
MR leave−one−out sensitivity analysis for
'finngen_R9_M13_OSTEOPOROSIS' on 'GCST90274771.tsv.gz'
